# Supplementary material for: Risk Factors and Outcomes of Recurrent Candidemia in Children: Relapse or Re-Infection?
Source: J Clin Med. 2019 Jan 16;8(1):99. doi: 10.3390/jcm8010099 (PMC6352033; doi:10.3390/jcm8010099)
Supplement: Supplementary file 1 [file jcm-08-00099-s001.pdf]

Supplementary Table 1. Comparisons of early recurrent episodes and late recurrent episodes of candidemia in children.

	Early recurrent episodes of candidemia (total n=29)	Late recurrent episodes of candidemia (total n=28)	P-value
Age			
Non-neonatal patients (years), median (IQR)	2.4 (0.9-7.2)	1.8 (0.8-7.1)	0.112
Neonatal patients (days), median (IQR)	53.5 (36.5-71.5)	66.5 (52.5-76.3)	0.223
Sex (male gender)	14 (48.3)	13 (46.4)	1.000
Hospital days until diagnosis, median (IQR)	41.0 (22.0-72.5)	44.0 (19.5-84.5)	0.451
Ward			0.062
Neonatal intensive care unit	7 (24.1)	5 (17.9)	
Pediatric intensive care unit	17 (58.6)	12 (42.9)	
Burn or surgical intensive care unit	2 (6.9)	3 (10.7)	
General wards	3 (10.3)	8 (28.6)	
Underlying chronic comorbidities[#]			
Congenital or genetic anomalies	6 (20.7)	5 (17.9)	0.786
Neurological sequelae	12 (41.4)	17 (60.7)	0.189
Cardiovascular disease	6 (20.7)	3 (10.7)	0.302
Chronic lung disease and/or pulmonary hypertension	13 (44.8)	6 (21.4)	0.092
Gastrointestinal sequelae	12 (41.4)	17 (60.7)	0.189
Renal insufficiency with/without dialysis	4 (13.8)	4 (14.3)	0.957
Hematological/Oncology cancer	4 (13.8)	0 (0)	0.060

Immunodeficiency	1 (3.4)	0 (0)	0.322
Autoimmune disease	1 (3.4)	0 (0)	0.322
Hepatic failure or cholestasis	0 (0)	1 (3.6)	0.305
Pathogens			0.679
Candida albicans	7 (24.1)	10 (35.7)	
Candida parapsilosis	13 (44.8)	10 (35.7)	
Candida tropicalis	1 (3.4)	2 (7.1)	
Candida glabrata	4 (13.8)	3 (10.7)	
Candida guilliermondii	0 (0)	1 (3.6)	
Other *Candida* spp.	4 (13.8)	2 (7.1)	
Clinical presentation			
Severe sepsis	15 (51.7)	10 (35.7)	0.289
Septic shock	12 (41.4)	8 (28.6)	0.408
Progressive and deteriorated[¶]	10 (34.5)	4 (14.3)	0.077
Disseminated candidiasis[$]	1 (3.4)	1 (3.6)	0.980
Predisposing risk factors[#]			
Receipt of systemic antibiotics[&]	29 (100)	27 (96.4)	0.491
Previous azole exposure[&]	15 (51.7)	9 (32.1)	0.182
Prior bacteremia[&]	25 (86.2)	21 (75.0)	0.331
Presence of CVC	29 (100)	28 (100)	1.000
Stay in an intensive care unit	14 (48.2)	16 (57.1)	0.526
Receipt of parenteral nutrition	21 (72.4)	21 (75.0)	0.981

Receipt of immunosuppressants	8 (27.6)	2 (7.1)	0.045
Artificial device other than CVC	20 (70.0)	21 (75.0)	0.770
Prior surgery[&]	12 (41.4)	7 (25.0)	0.263
Neutropenia (ANC< $0.5 \times 10^3/\mu L$)	11 (37.9)	6 (21.4)	0.248

[#]Indicated the presence of underlying condition or risk factor at onset of candidemia, and most patients with candidemia had >1 underlying condition and/or risk factor.

[&]Within one month prior to onset of candidemia, prior azoles exposure indicated patients received an azoles drug in addition to the antifungal agents at the time of candidemia.

[¶]Defined as candidemia episodes with more disseminated candidiasis and/or progressive multi-organ failure even after effective antifungal agents.

[$]Indicated positive *Candida* isolates recovered from more than two sterile sites, in addition to primary bloodstream infection.

IQR: interquartile range; CVC: central venous catheter; ANC: absolute neutrophil count.
